# Archives of cyanobacterial traits: insights from resurrected *Nodularia spumigena* from Baltic Sea sediments reveal a shift in temperature optima

**DOI:** 10.1093/ismeco/ycae140

**Published:** 2024-11-13

**Authors:** Cynthia Medwed, Ulf Karsten, Juliane Romahn, Jérôme Kaiser, Olaf Dellwig, Helge Arz, Anke Kremp

**Affiliations:** Leibniz Institute for Baltic Sea Research Warnemuende, Department of Biological Oceanography, Rostock, 18119, Germany; Applied Ecology and Phycology, Institute of Biological Sciences, University of Rostock, Rostock, 18059, Germany; Interdisciplinary Faculty, Department of Maritime Systems, University of Rostock, Rostock, 18059, Germany; Senckenberg Biodiversity and Climate Research Centre, Frankfurt am Main, 60325, Germany; Leibniz Institute for Baltic Sea Research Warnemuende, Department of Marine Geology, Rostock, 18119, Germany; Leibniz Institute for Baltic Sea Research Warnemuende, Department of Marine Geology, Rostock, 18119, Germany; Leibniz Institute for Baltic Sea Research Warnemuende, Department of Marine Geology, Rostock, 18119, Germany; Leibniz Institute for Baltic Sea Research Warnemuende, Department of Biological Oceanography, Rostock, 18119, Germany

**Keywords:** cyanobacteria blooms, Nodularia spumigena, resurrection approach, sediment archives, Baltic Sea, photosynthesis, traits

## Abstract

Cyanobacterial blooms in the Baltic Sea proliferated in recent decades due to rising sea surface temperatures, resulting in significant ecological impacts. To elucidate their current success, we examined ecophysiological, biochemical, and morphological traits of recent and ~33-year-old strains of *Nodularia spumigena* using a resurrection approach. The ability of many cyanobacteria to form dormant stages that can persist in anoxic sediments for decades provides a unique opportunity to study adaptive traits to past environmental conditions. A short sediment core from the Eastern Gotland Basin was processed to isolate strains of *N. spumigena* buried in 1987 ± 2 and 2020 ± 0.5 Common Era. Sequencing was used for species identification, followed by characterization of cell morphometry, carbon, nitrogen, and chlorophyll *a* content. Photosynthetic performance was evaluated by using pulse-amplitude modulated fluorimetry and oxygen optodes to assess light and temperature requirements. Our results revealed trait changes in *N. spumigena* over the past 3 decades: Temperature optimum for photosynthesis shifted from 15.3–21.1°C, which is consistent with the past and present local SST. Recent strains exhibited increased carbon, nitrogen, and chlorophyll *a* content despite decreased cell volume. The demonstrated adaptability of *N. spumigena* to increasing temperature suggests that this species will thrive in a warmer climate in the future. These insights will aid modeling efforts aimed at understanding and managing consequences of future cyanobacterial blooms in the Baltic Sea ecosystem.

## Introduction

The Baltic Sea is the largest brackish ecosystem on Earth, and recurring summer cyanobacterial blooms have been a natural phenomenon here since the beginning of the brackish water phase ~7500 years ago [1, 2]. Due to its shallow average depth of 52 m, the Baltic Sea is highly susceptible to climate-induced warming, resulting in a faster and stronger impact compared to the world’s oceans. The mean sea surface temperature (SST) in the Baltic Sea has increased by 1.35°C within the past 24 years, and it is predicted to rise further by 1.1°C to 3.2°C by the end of this century [3, 4].

Filamentous cyanobacteria, such as *N. spumigena* (Nostocales), play a crucial role as primary producers and drivers of biogeochemical cycles in the Baltic Sea, due to their ability of fixing nitrogen in specialized cells, heterocysts [5]. Several studies indicate that the intensity of cyanobacterial blooms in the Baltic Sea has increased since the 1960s, due to rising temperatures and changing nutrient conditions [6–8]. Rising intensity and frequency of cyanobacterial blooms can have far-reaching ecological and economic consequences for the respective ecosystem and coastal regions due to their potential toxicity for higher organisms, such as fish [9, 10].

Generally, cyanobacteria blooms are induced by calm weather conditions, sufficient light, ambient temperatures >16°C, and increased phosphorus concentrations in relation to nitrogen [11]. Aquatic cyanobacteria are expected to benefit from climate warming, as they generally exhibit—compared to eukaryotic microalgae—higher temperature requirements for physiological processes [12–14]. As a consequence, numerous cyanobacterial species are invading into warming aquatic habitats with negative consequences for the structure and function of native communities [15].

*N. spumigena* is well studied in terms of its potential toxicity, general occurrence, and nitrogen fixation in the Baltic Sea, particularly in the context of eutrophication and climate warming, but its ecophysiological characteristics, such as photosynthetic performance, are poorly understood [16]. Analysing such traits could provide valuable information on species-specific adaptation strategies and niche occupation in a temporally changing ecosystem. This information is crucial for understanding long-term changes in ecophysiological response patterns and making predictions for the future. Ecophysiological responses can change over time due to adaptive processes and may be linked to anthropogenic stressors, such as climate warming, as demonstrated for dinoflagellates [17]. Similar studies on bloom-forming cyanobacteria, however, are missing. Ecosystem scenario models often overlook potential long-term changes in cyanobacteria [14, 18] highlighting the need for further research in this area.

Under unfavorable conditions, *N. spumigena* forms resting stages (akinetes) that are an essential part of its life cycle, allowing survival under suboptimal conditions and serving as a dispersal propagule for colonization of new habitats [19]. Akinetes are densely packed with storage products, which increase weight and support cells, and eventually—after blooms—sink to the seafloor [20]. Here, resting stages accumulate and constitute seed banks that inoculate future blooms.

The Baltic Sea’s specific geographical and geological settings result in a limited supply of oxygenated saltwater from the North Sea. In combination with significant eutrophication, this leads to enhanced oxygen deficiency and permanent bottom water anoxia particularly in the deep basins but also in shallower coastal embayment and fjords [21]. Prevailing hypoxic to even sulphidic bottom waters prevent bioturbation, thereby favoring the preservation of organismal remains, including resting stages. Various organisms, including cyanobacteria and their akinetes, are deposited chronologically in undisturbed sediments, and may persist in respective sediment archives for decades and centuries before reactivation [22, 23]. Anoxic undisturbed bottom sediments of the Baltic Sea may serve as natural and precisely datable chronological archives of adaptation, which can provide insights into causal relationships between environmental change and trait evolution [24]. Resurrection of resting stages and subsequent trait characterization can be used to demonstrate that environmental conditions of the past led to cohort-specific adaptive changes, which might be reflected in infraspecific response patterns. So far, studies using this approach have primarily focused on dinoflagellates and diatoms [17, 24]. Recently, a study by Wood [25] provided compelling evidence of the resurrection potential of Baltic cyanobacteria.

In this study we investigated trait changes within Baltic *N. spumigena* populations over several decades by resurrecting past and present cohorts of akinetes from dated sediments of the Eastern Gotland Basin (EGB) that were deposited in 1987 ± 2 and 2020 ± 0.5 Common Era (CE). Both cohorts were compared in terms of their light and temperature requirements for photosynthesis, as well as their morphological and biochemical characteristics. We hypothesize that *N. spumigena* is ecologically successful in the Baltic Sea through adaptation to changing environmental conditions, specifically to higher water temperatures through development of temperature dependent traits.

## Material and methods

### Sediment sampling

**Figure 1:**
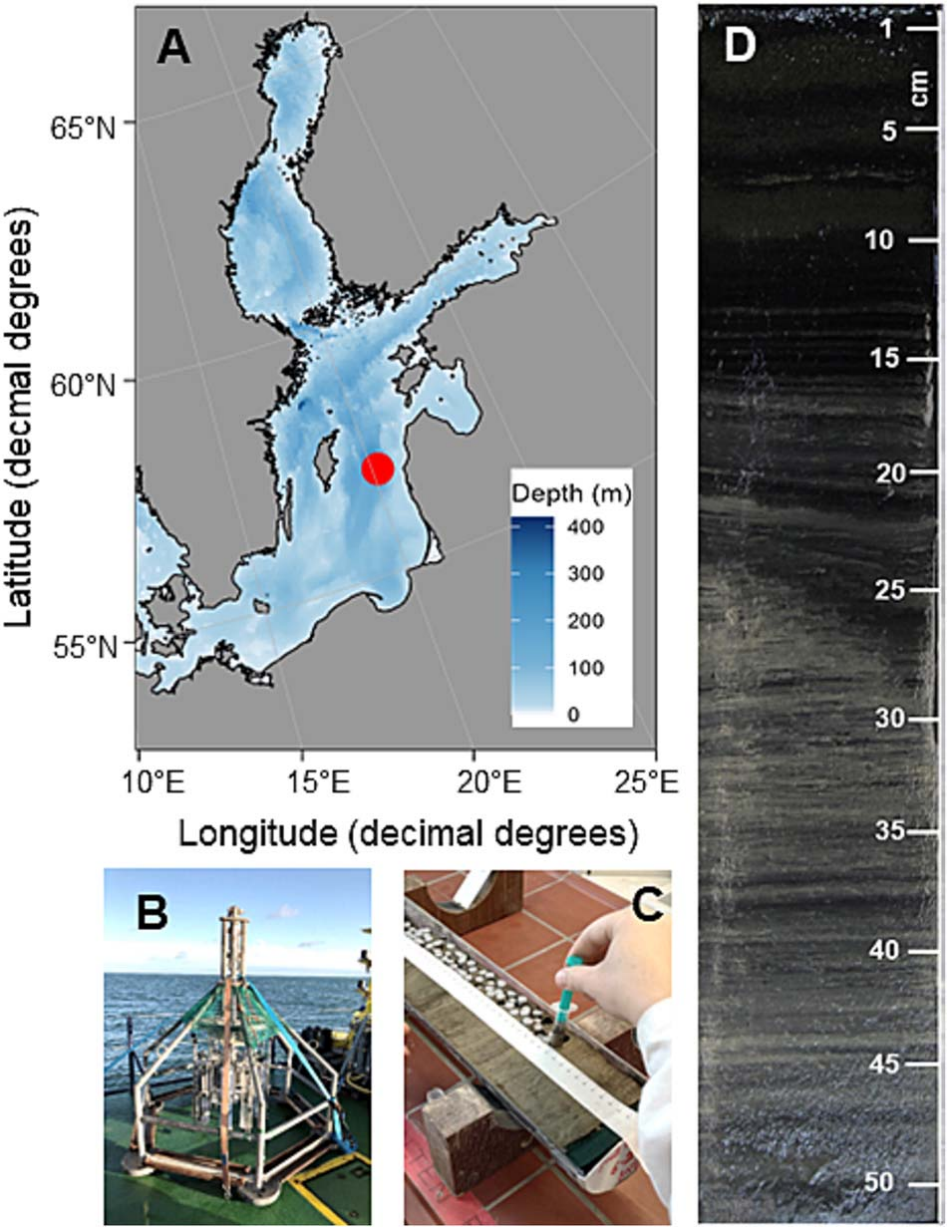
Sampling strategy: (A) Map of the Baltic Sea and cruise EMB262 with sampling location in the EGB (dot). (B) Multicorer. (C) Sliced core and sediment layer sampling for the age model with syringes. (D) Scan of core EMB262/6–28 from the EGB, 52 cm long (scale in cm).

A short sediment core (EMB262/6–28; 52 cm in length) was collected using a Multi Corer on the RV Elisabeth Mann Borgese cruise EMB262 in the EGB (57°17.004’ N, 020°07.244′ E, 241 m depth) in April 2021 (Fig. 1). This coring device is capable of recovering short cores with an undisturbed water–sediment interface. To avoid sediment alteration and disturbance, the core was immediately split lengthwise into two halves and further sampled on board [26]. The surface of one half-core was carefully cleaned and the outer sediment that came into contact with the core liner was omitted from sampling. For the resurrection experiments, ~6 mL of each sediment layer was sampled with a sterile syringe at 1–2 cm intervals and stored at 4°C in the dark.

### Sediment dating

Sediment dating is crucial to estimate the age of isolated and germinated cyanobacterial strains, as the age of the respective sediment layer is assumed to correspond to the year of akinete deposition. The age model of core EMB262/6-28 has been published in Schmidt [27].

The dating error has been estimated to ±2 years for 1987 CE (12 cm depth) and ± 0.5 years for 2020 CE (0 cm depth – core top).

### Summer surface temperature in the Eastern Gotland Basin from 1980–2022

Summer surface temperatures (June–August, 0–5 m depth) based on monitoring data by the Leibniz Institute for Baltic Sea Research (IOW, ODIN database) for the EGB was used to compare temperature-depended traits of the resurrected strains to past and current local conditions (Supplementary Fig. S1). Data representing 1987 ± 2 CE were obtained by averaging data from 1985–1988. Data representing 2020 ± 0.5 CE were obtained by using only the data from 2020.

### Resurrection, isolation, and culture conditions

**Figure 2:**
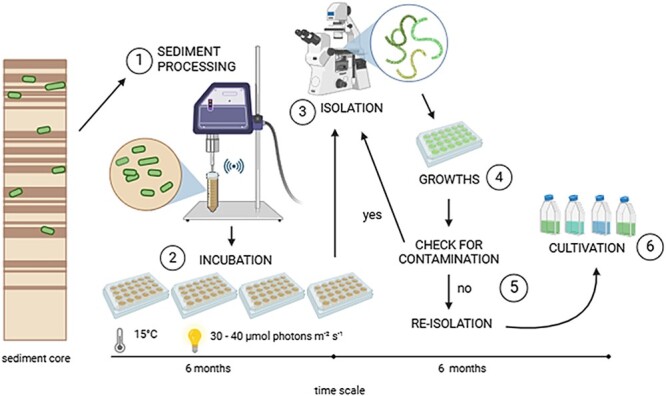
Schematic process of cyanobacteria resurrection from Baltic Sea sediment to clonal strains. Akinetes in the respective sediment core layer were suspended by using a sonicator [1]. Slurries were incubated in 24-well-plates for at least 6 months and samples were regularly checked for germination [2]. Germinated single filaments were isolated [3], incubated [4], and isolated again [5] to guarantee clonal strains. Established clonal cultures were kept at 15°C under low-light conditions [6]. Illustration created with BioRender.com.

In order to resurrect recent and subrecent *N. spumigena* strains, 15 layers were selected from the sediment samples of the core (Supplementary Table S1) and were carefully processed according to Legrand [23] and Wood [25] (Fig. 2). To avoid premature germination of the akinetes, samples were kept cool (4°C) and dark until processing. For each sediment slurry ca. 6 mL of wet sediment were transferred into a sterile 50 mL Falcon tube and resuspended in 45 mL sterile 7.5-PSU Baltic seawater. To separate the akinetes from sediment, the slurries were vortexed and then sonicated for 1 min at 80% intensity (constant cycle, Sonoplus HD70A, Bandelin, Berlin, Germany). The suspended sediment samples were cooled on ice in the dark, separated in two subsamples and diluted 1:1 (v/v) with cold sterile 7.5-PSU Baltic seawater, resulting in 100 mL of processed sediment slurry per layer in total. For each slurry, the germination experiments were performed in four 24-well-plates (96 replicates per sediment layer; tissue culture plate, non-treated, sterilized, VWR, USA). Wells were filled with 500 μL sediment slurry and 1 mL of prepared 7.5-PSU BG11_0_ according to Rippka [28] (Supplementary Table S2) and adjustments from Kuhl and Lorenzen [29] to minimize eukaryotic growth. The plates were sealed with parafilm and incubated at 15°C, 30–40 μmol photons m^−2^ s^−1^ (L36W/965, OSRAM, Germany) and a 16:8 h day-night-cycle for 6 months. All wells were checked regularly for germination under an inverted microscope (Axiovert 40C, ZEISS, Jena, Germany). When germination occurred in a well and filaments were morphologically identified as *N. spumigena* according to Komárek [30], only one filament was manually isolated by a micropipette from the respective well. This procedure was used to establish distinct strains germinating from disparate akinetes, which serve here as biological replicates from one age cohort. Species identities were later confirmed by molecular analysis. Isolated filaments were transferred into sealed 24-well-plates filled with BG11_0_ medium and incubated for 2 weeks at conditions described above. The isolation procedure was repeated to minimize contamination and ensure clonality of each strain. Once growing, strains were transferred into 50 mL culture flasks (Cellstar®, Greiner Bio-One GmbH, Frickenhausen, Germany) filled with fresh media and maintained as not axenic cultures. The following strains of *N. spumigena* were generated for the experiments and are maintained in the algal culture collection of the Institute for Baltic Sea Research, Warnemuende, Germany: from the top layer (1 cm, dated 2020 ± 0.5 CE) strain NSEGB1-2105, NSEGB1-2109, and NSEGB1-2111, from here on referred as recent strains 1-2105, 1-2109 and 1-2111. From 12 cm depth (dated 1987 ± 2 CE) NSEGB12-2101, NSEGB12-2102, and NSEGB12, from here on referred as subrecent strains 12-2101, 12-2102, and 12-2103.

### Molecular species identification

For molecular identification, isolated strains were sequenced for 16S according to Romahn [31]. Deoxyribonucleic acid (DNA) of ~1 mL concentrated fresh culture material was extracted using an Allprep DNA/ribonucleic acid (RNA) Mini Kit (QIAGEN). Cells were disrupted by a freeze–thaw process and the use of glass beads. Further purification was carried out according to the manufacturer’s protocol. Culture extracts were amplified with the metabarcoding primer Cya400 [32]. Due to culture contaminations of one sample with picocyanobacteria, two cultures were amplified again with the metabarcoding primer Cya_211XS [31], as they amplify less likely picocyanobacteria. For amplification, we used the AmpliTag Gold 360 Master Mix (Thermo Fisher, Waltham MA, USA). Polymerase chain reaction (PCR) products were purified with the PCR/DNA Purification Protocol (GENAXXON bioscience, Ulm, Germany) and Sanger sequenced at the Senckenberg BiK-F laboratory center (Frankfurt Main, Germany). For species identification, the sequences were blasted against the National Center for Biotechnology Information (NCBI) nt databases and phylogenetic trees were reconstructed from the 16S ribosomal RNA (rRNA) fragments with additional NCBI sequences for both the primer pair datasets (Supplementary Table S3). We confirmed all strains as *N. spumigena* by the combination of morphological and molecular data. A detailed methodological overview can be found in the supplements.

### Photosynthesis irradiance curves

Photosynthetic oxygen release rates of recent and subrecent *N. spumigena* strains were measured under increasing photon flux densities (PFD) to investigate the light requirements of the resurrected strains at a fixed temperature of 20°C. Therefore, eleven light levels were adjusted using neutral gray filter foils, which ranged from 0 to 1490 μmol photons m^−2^ s^−1^ (0, 4, 11, 27, 35, 89, 135, 336, 584, 886, 1490). The exact PFD was controlled inside each cuvette using a micro-sphere light meter (LI-250, LI-COR, Lincoln, United States) following Prelle [33].

Three technical replicates of each strain (each 3.1 mL) of log-phase suspension were filled into water-tempered cuvettes and each measured by using oxygen optodes (Fibox3, PreSens, Germany). To avoid carbon deficiency during measurements, sodium bicarbonate (NaHCO_3_, 2 mM final concentration) was added to each cuvette. Since *N. spumigena* are capable of buoyancy, a homogeneous light field was difficult to establish. Therefore, before each new light step, the cuvettes were carefully shaken until the filaments were evenly distributed again in the water column, supported by a small magnetic stirrer. After the experiment, total chlorophyll *a* per sample was determined. Therefore, each recorded *N. spumigena* suspension from each cuvette was filtered onto individual GF/6 glass fiber filters (Ø 25 mm, Whatman, UK). Chlorophyll *a* was extracted as described above and calculated according to Ritchie [34]. The oxygen production per PFD and time was normalized to the amount of total chlorophyll *a* per sample, and the PI-curves and the resulting parameters were fitted and calculated after Walsby [35]. Photosynthetic parameters were estimated by fitting least-square regression curves to the measured values using the solver function in MS Office Excel 2016. The resulting PI-curves were used to calculate the maximum rate of net primary production (NPP_max_), respiration (R), light saturation point (I_k_), light compensation point (I_c_), light utilization coefficient (α), and photoinhibition coefficient (β).

### Photosynthesis and respiration at increasing temperature

Photosynthetic oxygen production and respiratory oxygen consumption of recent and subrecent *N. spumigena* strains were measured under increasing temperature in 5°C increments from 5 to 40°C to investigate the temperature requirements for photosynthesis and respiration. The same oxygen optode system as used for the PI-curves described above was used. The temperature measurements were performed at constant and saturating light level of 300 μmol photons m^−2^ s^−1^ and with three technical replicates per strain according the approach of Karsten [36]. At each temperature step (see above), always 10 min dark phase (respiration) were followed by always 20 min light phase (photosynthesis). Temperature optimum (T_opt_), Temperature maximum (T_max_), and maximum oxygen production (P_max_) were calculated according to Blanchard [37] by using R (version 4.3.1).

### Characterization of recent and subrecent *Nodularia spumigena* traits

For morphological, biochemical and ecophysiological trait characterization, the six resurrected strains were grown in 50 mL culture flasks at culture conditions described before. Growth was monitored based on phycocyanin fluorescence (excitation: 590 nm, emission 650 nm) by using a microplate reader (Infinite® 200 PRO, TECAN, Männedorf, Switzerland) as preliminary experiments showed a positive correlation between cell counts, chlorophyll *a* fluorescence, and optical density [38, 39]. Samplings for each trait were carried out with log-phase cultures of each strain.

#### Morphology

To characterize cell sizes of all three cell types (vegetative cells, heterocysts, and akinetes) as age-specific morphological trait, 1 mL of each strain culture were fixed with acidic 1% Lugol solution. Each sample was evaluated in an Utermöhl-chamber (HYDRO-BIOS, Kiel, Germany) by using an inverted microscope (Axiovert S100, ZEISS, Jena, Germany). Lengths and widths of 20 cells from different filaments per cell type were measured in all six strains using the software OLYMPUS Stream Essentials 2.4. Cell volumes of vegetative cells and heterocysts were calculated according to the geometric form of a cylinder [40]. For the akinetes the formula of an ellipsoid (V = 4πabc/3) was used.

#### Photosynthetic performance using chlorophyll a fluorescence

To evaluate the physiological state and the activity of photosystem II (PSII), the effective quantum yield Y(II) was measured by using a pulse amplitude modulated fluorometer (WinPAM 2500, Walz, Germany). For every strain, 4 × 100 μL of each culture were transferred to GF6 filter (Ø 47 mm diameter, Whatman, UK) arranged as dots. These filters were placed on a polystyrol box with 0.8 cm distance between samples and PAM fiber optic. Y(II) was measured and calculated using the equations of Genty [41].

#### Chlorophyll a and carbon, nitrogen content

Before analysis of chlorophyll *a* as well as cellular carbon (C) and nitrogen (N) content, log-phase cultures of recent and subrecent strains were washed through a 30 μm gauze to minimize associated bacteria. Then the filaments of each strain were transferred to a Falcon tube filled with 40 mL 7.5-PSU Baltic seawater. From this culture solution samples for chlorophyll *a* and C/N analysis were taken as follows:

To determine chlorophyll *a* content, 3 × 5 mL of each culture solution (three technical replicates per strain) were filtered through a GF/F filter (25 mm diameter, Whatman) by using a vacuum pump. Each filter was transferred into a 15 mL Falcon tube and frozen at −20°C. For chlorophyll *a* extraction, 10 mL of 96% ethanol were added to each tube, vortexed for 10 s and incubated at 70°C for 20 min. The samples were then cooled on ice and centrifuged for 10 min at 1.800 x *g* at 5°C to decrease turbidity. Chlorophyll *a* measurements and calculations were performed photometrically after Ritchie [34].

For C/N content, again 3 x 5 mL of each culture solution were filtered through pre-weighted GF/F filter (Ø 25 mm, Whatman). The filters were then dried and weighted again to determine the dry weight. Total carbon and nitrogen (C_t_ and N_t_) were determined by dry combustion using an elemental analyser (UNICUBE® Elementar Analysensysteme GmbH, Langenselbold, Germany).

### Statistical analysis

The data were analysed for significant differences in traits between the two age cohorts of *N. spumigena* by using *t*-test, non-normally distributed data were analysed using the Wilcoxon test by using R (version 4.3.1). For the oxygen data, outlier values that originated from air bubble formation in the cuvette were deleted before statistical analysis.

## Results

### Germination success of *Nodularia spumigena* from the Eastern Gotland Basin

Resurrection experiments yielded 13 strains of *N. spumigena* (Supplementary Table S1). The surface layer (estimated age: 2020 ± 0.5 CE) is represented by 10 different strains of *N. spumigena* that germinated after 2–3 weeks of incubation, of which three were selected for later experiments. From the 12 cm depths layer (estimated age: 1987 ± 2 CE) only three different strains had germinated after 6–8 weeks of incubation. No additional *N. spumigena* strains germinated in any of the investigated sediments layers after this time interval. Molecular and morphological analysis confirmed identity of *N. spumigena* for all the six strains (see supplements: Supplementary Figs S2 and S3 and detailed molecular results).

### Light-dependent photosynthesis

**Figure 3:**
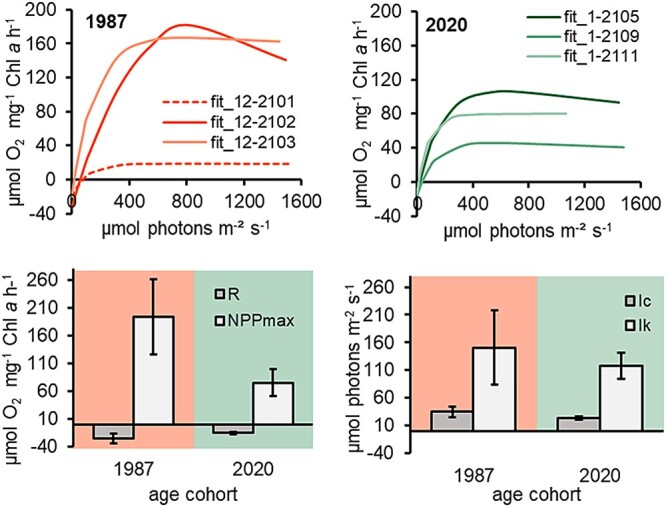
Results of photosynthetic oxygen release rates (PI-curves)—measured by oxygen production with optodes. Upper plots: fitted photosynthesis rates (μmol O_2_ mg^−1^ Chl a h^−1^) in relation to increasing photon flux density (μmol photons m^−2^ s^−1^) of all resurrected recent (2020 ± 0.5 CE) and subrecent (1987 ± 2 CE, dotted line = 12-2101) *Nodularia spumigena* strains. PI-curves were fitted according to Walsby [35]. From PI-curves, photosynthetic parameters (NPP_max_ = photosynthetic maximum, I_k_ = light saturation point, I_c_ = light compensation point, R = respiration rate) were calculated according to Walsby *et al.* 1997 (except for 12-2101). Lower plots: mean ± standard derivation of respective photosynthetic parameters according to age cohort (*n* = 3). Strains were kept at 15°C in 7.5-PSU BG11_0_ medium.

All resurrected *N. spumigena* strains exhibited photosynthetic oxygen production rates with increasing photon flux up to 1490 μmol photons m^−2^ s^−1^ (Fig. 3). Respiration in the dark was low for all strains, indicating low oxygen consumption by associated bacteria. In none of the strains except 12-2102, photoinhibition was observed, even at high PFDs. For all strains except 12-2101 the characteristic PI-curve parameters were calculated according to Walsby [35] (Supplementary Table S4). Unfortunately, the strain 12-2101 did not succeed in this experiment. Overall, calculated photosynthetic parameters showed no significant differences in the light requirements between the recent and the subrecent strains (Supplementary Table S4). The highest and lowest NPP_max_ values were found in the subrecent strains. The light compensation point (I_c_) varied between 17 and 90 μmol photons m^−2^ s^−1^ for all studied *N. spumigena* strains, the highest values were obtained from two subrecent strains (Fig. 3). The light saturation point (I_k_) was on average 118 μmol photons m^−2^ s^−1^ for the recent strains and 131 μmol photons m^−2^ s^−1^ for the subrecent strains.

### Temperature-dependent photosynthesis and respiration

**Figure 4:**
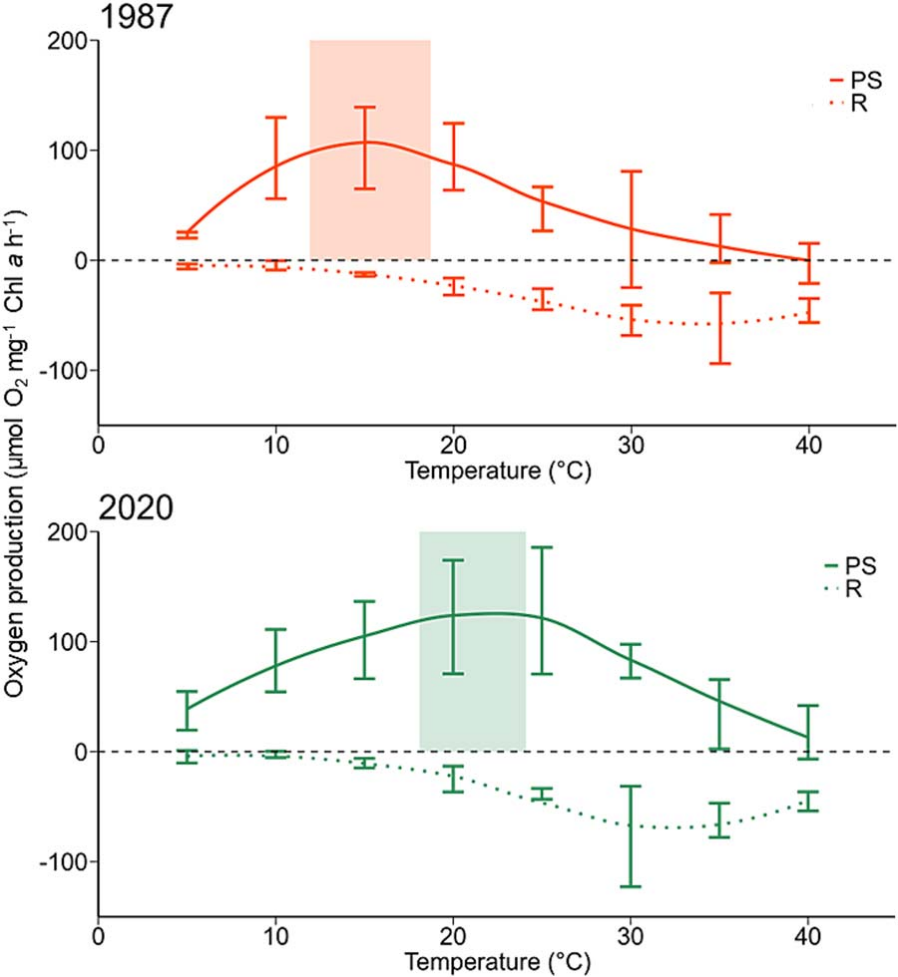
Mean photosynthetic oxygen production and mean respiratory consumption at increasing temperature (5–40°C) of the resurrected recent (2020 ± 0.5 CE, *n* = 3) and subrecent (1987 ± 2 CE, *n* = 3) *N. spumigena* strains in μmol O_2_ mg^−1^ Chl *a* h^−1^. Means (smoothed line) ± standard derivation (error bars) of oxygen production (solid line, PS = photosynthesis) during irradiance at 300 μmol photons m^−2^ s^−1^ and oxygen consumption in dark (dotted line, R = respiration). The transparent rectangles represent the calculated range (mean ± standard derivation) of optimal photosynthesis (for details, see Table 1).

All strains showed photosynthetic activity under optimum irradiance conditions (300 μmol photons m^−2^ s^−1^) and respiration rates in the dark, which indicates that the resurrection approach did not influence viability of both physiological processes. Recent and subrecent strains exhibited clear differences in maximum oxygen production and in optimum temperature for photosynthesis (Fig. 4 and Table 1). In the recent strains, mean P_max_ was significantly higher compared to the subrecent strains, but with a standard derivation of 40.5 μmol O_2_ mg^−1^ Chl *a* h^−1^ indicating strain-specific variations (Table 1). The calculation of the optimum temperature (T_opt_) for photosynthesis revealed a significant difference between recent and subrecent strains. For *N. spumigena,* T_opt_ shifted from 15.3°C in 1987 ± 2 CE to 21.1°C in 2020 ± 0.5 CE, i.e. by 5.8°C in 33 years (Fig. 4, Table 1). The recent strains showed a wide temperature range from 10°C to <25°C, where 50% of P_max_ were reached. Two of the recent strains even exhibited a P_max_ of 70% of the optimum at 30°C. The subrecent strains displayed a smaller range from only 10°C to 20°C and P_max_ decreased abruptly to <43% as soon as a temperature ~20°C was reached. The maximum temperature for photosynthesis (T_max_) is consistent across recent and subrecent strains species, averaging 37.9 ± 1.9°C. A small or even no respiration was observed at temperatures of 5°C and 10°C in the subrecent strains, while the recent strains exhibited respiratory performance at these low temperatures. The maximum level of respiration was observed between 30°C and 35°C for all strains.

**Table 1:** Temperature optimum (T_opt_) for photosynthesis, photosynthetic temperature maximum (T_max_) in °C, and maximum photosynthetic oxygen production (P_max_) in μmol O_2_ mg^−1^ Chl *a* h^−1^ of resurrected recent and subrecent *Nodularia spumigena* strains (age cohort in bold) calculated according to Blanchard [37].

Age cohort	T_opt_ (°C)	P_max_ (μmol O_2_ mg^−1^ Chl *a* h^−1^)	T_max_ (°C)
**2020 ± 0.5** **CE**	21.1 ± 3.0	119.6 ± 40.5	38.7 ± 8.6
**1987 ± 2 CE**	15.3 ± 3.4	83.5 ± 25.9	37.2 ± 1.7
*P*-value	^**^0.002	^*^0.038	0.138

Significant values indicated as asterisk: ^*^P < .05, ^*^^*^P < .005, ^*^^*^^*^P < .0005.

### Morphological characteristics

**Figure 5:**
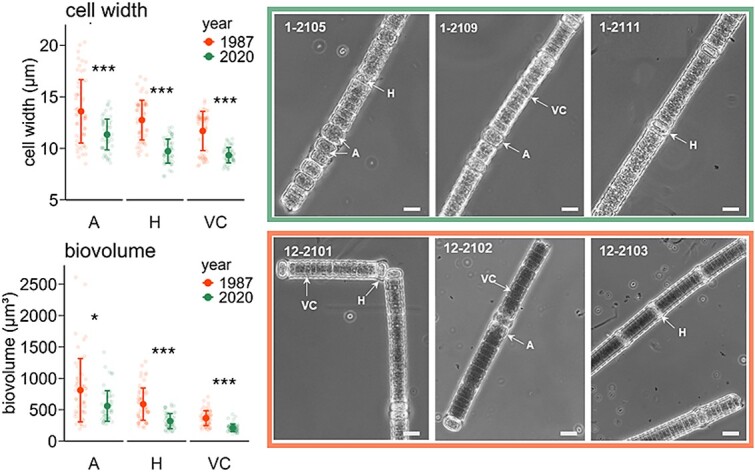
Morphological characteristics and microscopic view of resurrected recent (2020 ± 0.5 CE) and subrecent *N. spumigena* (1987 ± 2 CE). Left: mean ± standard derivation of the calculated cell width (μm) and biovolume (μm^3^) of each cell types according to the age cohort (= year of deposition). Right: Microscopy images from selected *N. Spumigena* strains. VC = vegetative cells, H = heterocysts, a = akinetes. Scale bare = 10 μm. Statistical differences between the temporal cohorts were tested by using *t*-test/ Wilcoxon test (*n* = 3). Significant values indicated as asterisk: ^*^P < .05, ^**^P < .005, ^***^P < .0005.

Macroscopically, the filaments of the resurrected *N. spumigena* presented a typical dark green coloration in the culture flasks. Single filaments exhibited a brownish color under the microscope. All strains revealed the ability of buoyancy in culture. Vegetative cells and heterocysts (H) were flat cylindrical, whereas the akinetes (A) were spherical to oval (Fig. 5). Heterocysts were 1 μm longer and akinetes 2.5 times longer than vegetative cells in all strains. The mean cell lengths of heterocysts and akinetes of the recent strains showed almost identical values compared to the cell lengths of the subrecent strains (Supplementary Table S5). The mean lengths of the vegetative cells between the age cohorts exhibited significant differences with a mean value of 3.06 ± 0.63 μm for the recent strains and 3.38 ± 0.60 μm for the subrecent strains. All cell types of the strains from 1987 ± 2 CE were significantly wider (2–3 um) than those of the 2020 ± 0.5 CE (Fig. 5 and Supplementary Table S5). Vegetative cells from subrecent strains had a mean width of 11.7 ± 1.9 μm compared to 9.3 ± 0.7 μm in recent strains. Heterocysts and akinetes of the subrecent strains were also significantly wider (H: 12.7 ± 1.9 μm; A: 13.6 ± 3.1 μm) and revealed a higher range in cell widths than cells from the recent strains (H: 9.7 ± 1.2 μm; A: 11.3 ± 1.5 μm). Consequently, the mean biovolume of all cell types were significantly lower in the recent strains than in the subrecent strains (Fig. 5).

### Photosynthetic efficiency

**Figure 6:**
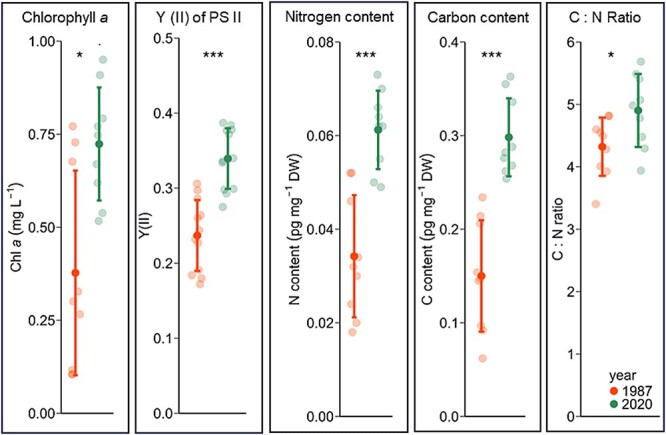
Means and standard derivation of biochemical parameters and photosynthetic performance in recent (2020 ± 0.5 CE) and subrecent strains (1987 ± 2 CE) of resurrected *N. spumigena* (*n* = 3). Y(II) of PS II = effective quantum yield of PSII, DW = dry weight. Significant values resulting from *t*-test/ Wilcoxon test are indicated as asterisks: ^*^P < .05, ^**^P < .005, ^***^P < .0005.

The mean Y(II) was significantly higher in the recent strains [Y(II) = 0.34 ± 0.04] than in the subrecent strains [Y(II) = 0.24 ± 0.05; *P* = 1.05E-05; Fig. 6, Supplementary Table S6]. The highest Y(II) was reached in the recent strain 1–2109 (0.39 ± 0.02) and the lowest in the subrecent strain 12-2102 *(*0.18 ± 0.01).

### Chlorophyll *a* and C/N

The mean chlorophyll *a* amount in the recent strains was 0.72 ± 0.14 mg L^−1^, which is significantly higher than in the subrecent strains with a mean of 0.38 ± 0.26 (*P* = .006; Fig. 6, Supplementary Table S6). The mean carbon content in the recent strains was 0.3 ± 0.04 pg mg^−1^ DW, twice as high as in the subrecent strains (0.15 ± 0.06 pg mg^−1^ DW, *P* = 2.38E^−05^). Also, the mean nitrogen content in the recent strains (0.06 ± 0.0 pg mg^−1^ DW) was twice as high as in the subrecent strains (0.03 ± 0.01 pg mg^−1^ DW, *P* = 1.41E^−04^). The mean C:N ratio was significantly higher in the recent strains (4.9 ± 0.55) than in the subrecent strains (4.32 ± 0.44, *P* = .033; Fig. 6 and Supplementary Table S6).

## Discussion

In this study we resurrected cyanobacteria akinetes and subsequently compared morphological, biochemical and ecophysiological traits of recent (2020 ± 0.5 CE) and subrecent (1987 ± 2 CE) strains of *N. spumigena*, which to our knowledge was done here for the first time. Photosynthetic parameters showed high activity of resurrected strains, indicating that resurrection had no negative effect on photosynthesis of both cohorts. There was a strong shift in the temperature optima for photosynthesis from 15.3°C to 21.1°C, indicating a significant photosynthetic adaptation within 33 years of climate warming. In contrast, no differences in photosynthetic light requirements could be observed. Cells of 1987-strains were larger compared to the 2020-strains. Chlorophyll *a* content, Y (II), as well as carbon and nitrogen content and C:N ratio were higher in the recent strains.

As photosynthesis is a temperature-dependent process due to the many enzymatic steps involved, and as summer SST increased in the EGB by ~6°C in 33 years, differences in the photosynthetic response to temperature between recent and subrecent *N. spumigena* were expected. Oxygen optode measurements indicate that the temperature optimum for photosynthesis had shifted by 5.8°C over the past 33 years (Fig. 4). The observed T_opt_ values in both age cohorts are in close agreement with the prevailing mean summer temperatures ~1987 and 2020 (Supplementary Fig. S1). This suggests that *N. spumigena* adapted to the recent and past local temperature conditions. Moreover, these differences in optimal temperature could be attributed to increased frequency in the occurrence of summer heat events [42], such as in 2003 [43] and 2018 [44] and the rise in record-breaking summer SSTs [45]. These altered temperature conditions in the Baltic Sea over the past decades could have facilitated natural selection of *N. spumigena* strains, which have the ability to perform photosynthesis more effectively at higher temperatures. Cyanobacteria in general have high strain-specific response patterns to environmental conditions [46, 47]—a feature that could increase the success of the selection process and, therefore, the survival of the species. Furthermore, proteins and fatty acids are strongly affected by temperature changes. Selected strains of *N. spumigena* probably exhibit a more efficient and faster change of the fatty acid composition in the thylakoid membranes compared to less temperature tolerant strains, since temperature changes primarily affects membrane fluidity [48]. As the photosystems in cyanobacteria are located on the thylakoid membrane, any changes in its fluidity have consequences for the photosynthesis. In addition, cyanobacteria respond to temperature changes by synthesizing chaperones and heat shock proteins that assist protein folding under heat stress [49]. Strains that function more effectively in these processes would have an advantage during temperature fluctuations and increasing heat events.

It is generally assumed that cyanobacteria benefit from an increase in SST due to their competitive advantage under warmer conditions compared to eukaryotic phytoplankton [13, 50]. Our data support this assumption, as temperatures between 15°C and 25°C led to at least 70% of the maximum photosynthetic oxygen production in the recent strains from 2020 ± 0.5 CE. Such a wide temperature tolerance range was recently confirmed for *N. spumigena*, based on long-term monitoring data [51]. The authors defined a temperature range of 16.5–26.7°C as the ecological niche of *N. spumigena.* These data not only align with our finding of higher temperature optima, but also confirm the suitability of the resurrection approach. Older observations generally report that *Nodularia* blooms proliferation occurred at temperatures <20°C [52, 53]. In contrast, our study showed a photosynthetic optimum for recent *N. spumigena* at 21.1°C, which agrees very well with the calculated optimum of 21.6°C by Telesh [51]. However, our results also indicate that *N. spumigena* was able to adapt its photosynthetic optimum to the local conditions in the EGB then and now. Furthermore, recent *N. spumigena* strains exhibit a tendency to maintain at least 50% photosynthetic efficiency even at temperatures up to 30°C. In the face of global warming, this characteristic promotes survival. Significant increase in photosynthetic temperature optimum by 5.8°C over the past 33 years, along with the observed trends in recent strains to tolerate even higher temperatures, points to continuing adaption to rising temperature in the future. Consequently, further increases in SSTs and a rise in heat events in the near future [44] will probably favor the proliferation of this species, in the Baltic Sea. Concerning the temperature factor, our results indicate that *Nodularia* will continue to be present in the Baltic Sea during the summer months in the future.

The P-I-curves and calculated I_k_ and I_c_ values exhibited no differences in light requirements between the recent and subrecent strains. The light conditions in the Baltic Sea are characterized by strong fluctuations in the PFDs due to the rapid changes in hydrological and meteorological conditions, such as cloud coverage. We do not expect changes in the light conditions between 1987 ± 2 CE and 2020 ± 0.5 CE, which is supported by nearly identical PI-curves of both age cohorts of *N. spumigena*. Nevertheless, these results contribute to a better understanding of the obvious success of *N. spumigena* during the summer months: The detected low I_k_ and I_c_ are sufficient for positive net photosynthesis, particularly on cloudy days in the Baltic Sea. Comparable I_k_ and I_c_ values were measured by Stal and Walsby [54] on other Baltic *N. spumigena* isolates. In addition, the studied *N. spumigena* strains showed minor to no photoinhibition at enhanced PFD (~1490 μmol photons m^−2^ s^−1^), pointing to high photophysiological plasticity. Reduced photoinhibition guarantees photosynthesis and growth under high light conditions, which is advantageous for *N. spumigena* and it enables the species to remain at the water surface and thus outcompete other phytoplankton species.

Cell size in phytoplankton is closely related to environmental changes in e.g. temperature, nutrient supply, and light conditions [55, 56]. The cells of recent *N. spumigena* strains were 41–46% smaller than those from the 1987-strains, which resulted mainly from a reduction in the lateral dimension. This could be a result of the increase in SST by ~6°C in the EGB (Supplementary Fig. S1) since 1980, as warmer temperatures are associated with smaller body sizes [57]. Warmer temperatures further increase all metabolic rates in cells and hence can affect the membrane fluidity by changes in fatty acid composition [58]. Chen [59] reported smaller cell sizes in *Microcystis aeruginosa* at 25°C compared to 15°C. A further reason for the observed reduction in cell size in recent strains may also be related to the lower phosphate ratios in the Baltic Sea today (2020) compared to the 1980s [60]. Cell sizes effect the expansion of blooms, and also grazing and changes in this trait can thus indirectly influence the composition of the food web [56]. Therefore, further investigation into cell size changes in response to environmental changes is needed. Furthermore, the observed differences in cell size may serve as an initial indicator of the adaptive potential of *N. spumigena* in this trait to local temperature and nutrient conditions. Moreover, the biochemical traits differed significantly between both age cohorts, but further research is needed to evaluate whether this is more due to intraspecific variations than trait adaptation to specific environmental conditions.

In summary, we present, for the first time, a trait comparison of recent and 33-year-old cohorts of Baltic *N. spumigena*. The main result was a 5.8°C shift in the temperature optimum between subrecent and recent strains of the species. The measured optima correspond very well to the mean summer SST of the respective time, which strongly implies that *N. spumigena* is able to rapidly adapt to temperature increase as presented by current global warming. Further, we found that recent and subrecent *N. spumigena* differed in both biochemical and morphological traits, which likely are attributed to changed temperature and nutrient conditions of the past three decades. Nevertheless, future studies need to determine to what extent such indicated shifts represent long standing trends and/or natural variability. Our results provide a basis for future modeling of cyanobacterial bloom development in the Baltic Sea, which were so far not considered in existing models.

## Supplementary Material

Supplementary_material_medwed_et_al_revised_final_ycae140

## Data Availability

All data generated or analysed during this study are included in this published article and its supplementary information files. The sequences generated during the current study are available in NCBI. Details and accession numbers are provided in Supplementary Table S3.
