# Supplementary material for: Archives of cyanobacterial traits: insights from resurrected Nodularia spumigena from Baltic Sea sediments reveal a shift in temperature optima
Source: ISME Commun. 2024 Nov 13;4(1):ycae140. doi: 10.1093/ismeco/ycae140 (PMC11605557; doi:10.1093/ismeco/ycae140)
Supplement: Supplementary_material_medwed_et_al_revised_final_ycae140 [file supplementary_material_medwed_et_al_revised_final_ycae140.pdf]

# - *Supplementary material* -

Archives of Cyanobacterial Traits: Insights from resurrected *Nodularia spumigena* from Baltic Sea Sediments reveal a shift in Temperature Optima

Cynthia Medwed[1], Ulf Karsten[2,4], Juliane Romahn[3], Jérôme Kaiser[1], Olaf Dellwig[1], Helge Arz[1], Anke Kremp[1]

[1] Leibniz Institute for Baltic Sea Research Warnemuende, Rostock, Germany

[2] Applied Ecology and Phycology, Institute of Biological Sciences, University of Rostock, Rostock, Germany

[3] Senckenberg Biodiversity and Climate Research Centre, Frankfurt am Main, Germany

[4] Interdisciplinary Faculty, Department of Maritime Systems, University of Rostock, Rostock, Germany

corresponding author: cynthia.medwed@io-warnemuende.de

Table S1: Overview of the chosen and tested sediment layers from the core of the EGB and results of the resurrection experiment. Green = germination of recent *N. spumigena* strains (2020 ± 0.5 CE), orange = germination of subrecent *N. spumigena* strains (1987 ± 2 CE). Three out of 10 recent strains were selected for later analysis due to non or minor contamination of bacteria and fungi.

**selected sediment layers (cm depths)**	**germinated strains of *N. spumigena***
1 (top)	10
3	0
12	3
14	0
15	0
17	0
20	0
22	0
23	0
24	0
37	0
38	0
46	0
47	0
51	0

Figure S1: Temperature conditions for 0-5 m water depths from 1980 – 2022 for the eastern Gotland Basin, Baltic Sea. Orange rectangle: marks the period of origin of the strains originate from 1987 ± 2 CE; Green rectangle: marks the period of origin of the strains originate from 2020 ± 0.5 CE. Data were conducted from monitoring data from ODIN data base of the Leibniz Institute for Baltic Sea Research, Germany.

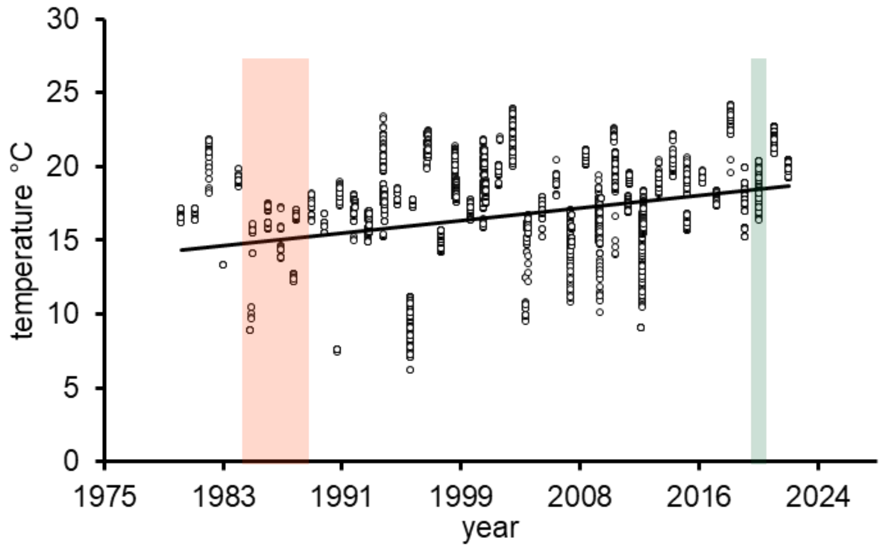

Table S2: Recipe for BG11$_0$ medium after Rippka 1992.

BG11$_0$ (= BG11 without NaNO3) after Rippka 1992
add 1 mL of each Stock solution to 992 mL of autoklaved 0.2 µm-filtered Sea water

chemicals	Stock solution g/100 mL de-ionized water
$K_2HPO_4 \times 3\ H_2O$	4
$MgSO_4 \times 7\ H_2O$	7.5
$CaCl_2 \times 2H_2O$	3.6
citric acid	0.6
ammonium ferric citrate	0.6
EDTA	0.1
Na_2CO_3	2

+ 1mL micronutrient solution*

* Composition of the micronutrient solution (after Kuhl and Lorenzen 1964):

chemicals	mg/ 100 mL de-ionized water
H_3BO_3	6.1
$MnSO_4$	16.9
$ZnSO_4 \times 7\ H_2O$	28.7
$CuSO_4 \times 5\ H_2O$	0.25
$(NH_4)6Mo_7O_{24} \times 4\ H_2O$	1.25

Suppl. Methods:

detailed description for molecular species identification:

For genetic species identification, the isolated strains were analysed by **16S rRNA fragment sequencing**. DNA of approx. 1 mL concentrated (floating biomass on the medium surface) fresh culture material by was extracted by using an Allprep DNA/RNA Mini Kit (QIAGEN). To disrupt the cells, the samples were treated 10 times alternately for 1 min in liquid nitrogen and then for 2 min at 65°C in a thermomixer (Eppendorf). Afterwards, approx. 10 - 20 0.1 mm glass beats and 500 µL RLT buffer (RNeasy mix + ß-Mercaptoethanol 100:1, QIAGEN) was added to each sample and grinded for 5 min at an intensity of 10 (Vortex Genie 2, MoBIO, USA). The extracts were then centrifuged at 10,000 x *g* for 1 min (mini spin plus, Eppendorf) and the supernatants transferred to the AllPrep DNA spin column from the kit. Further purification was carried out according to the manufacturer's protocol.

Culture extracts were **amplified with the metabarcoding primer** Cya400 (F: 5'-GGGGAATYTTCCGCAATGGG-3', R: 5'-CTACWGGGGTATCTAATCCC-3', Nübel et al.

(1997). We identified contamination in the Nodularia sp. 1-2105.1 with picocyanobacteria with the primer pair Cya400. Therefore, *Nodularia* sp. 1-2105.1 as well as the *Nodularia* sp. 1-2109 for comparability were amplified again with the primer pair Cya_211XS. Both DNA primer pairs amplify the same region. However, Cya_211XS appear to amplify less likely picocyanobacterial (Romahn et al. 2024, under revision). For amplification, we used the AmpliTag Gold 360 Master Mix (Thermo Fisher, Waltham MA, USA). The protocol included the following reagent ratio of 12.5 µL Mastermix, 9 µL water, 2.5 µL sample and 1 µL primer pair (10 µM stock). The **cycling program** included an initial denaturation step at 94°C for 5 min, primer dependent cycles of 94°C for 30 sec, primer dependent annealing temperature for 30 sec, 68°C for 45 sec and a final elongation step at 72°C for 10 min. We used 30 cycles and an annealing temperature of 60°C for Cya400 and 34 cycles and an annealing temperature of 58.8°C for Cya_211XS. PCR products were purified with the PCR DNA Purification Protocol (GENAXXON bioscience, Ulm, Germany) and Sanger sequenced at the Senckenberg BiK-F laboratory center (Frankfurt Main, Germany).

Forward and reverse sequences of each strain were trimmed with a cut off error probability limit of 5% and merged them via Geneious Prime (2023.2.1). **For species identification**, the sequences were blasted against the NCBI nt databases and phylogenetic tree of 16S rRNA fragments for both primer pair datasets reconstructed. Therefore, we downloaded sequences of *Nodularia* species and other Nostocalean species as outgroups with primary focus on Baltic species from NCBI (suppl Table S6) (Moffitt et al. 2001, Wood et al. 2021, Pardo-De la Hoz et al. 2023). All 16S rRNA sequences were aligned using MAFFT based on local pairwise alignment and at maximum 1000 iterations (v7.481) (Katoh & Standley, 2013). Sequences were cut into multiple sequence alignments of the same length in Geneious Prime and tree was reconstructed with ultrafast bootstrap of 1000 replicates for each dataset using IQTREE2 (v 2.1.3, Schmidt & von Haeseler 2015). The best-fit model was automatically chosen by ModelFinder according to BIC. The short fragment tree was based on K2P+I+G4 model and the long fragment tree was based on TIM2+F+R2 model. We conducted the tree reconstruction four times for both dataset and the tree with lowest BIC value was chosen in each case. The phylogenetic tree was rerooted and visualised in R (4.3.2) using the r packages ggtree (v v3.10.1, Yu et al. 2017) and treeio (v1.26.0, Wang et al. 2020). We collapsed nodes with a bootstrap value below 50. The phylogenetic trees were outgroup-rooted using Dolichospermum/ Aphanizomenon for the short fragment tree and Cyanobium/ Synechococcus as outgroup.

references:

Moffitt, M. C., Blackburn, S. I., & Neilan, B. A. (2001). rRNA sequences reflect the ecophysiology and define the toxic cyanobacteria of the genus Nodularia. *International Journal of Systematic and Evolutionary Microbiology*, *51*(2), 505-512.

Pardo-De la Hoz, C. J., Magain, N., Piatkowski, B., Cornet, L., Dal Forno, M., Carbone, I., ... & Lutzoni, F. (2023). Ancient rapid radiation explains most conflicts among gene trees and well-supported phylogenomic trees of Nostocalean cyanobacteria. *Systematic biology*, *72*(3), 694-712.
Wood, S. M., Kremp, A., Savela, H., Akter, S., Vartti, V. P., Saarni, S., & Suikkanen, S. (2021). Cyanobacterial akinete distribution, viability, and cyanotoxin records in sediment archives from the Northern Baltic Sea. *Frontiers in Microbiology*, *12*, 681881.
Katoh, K., & Standley, D. M. (2013). MAFFT multiple sequence alignment software version 7: improvements in performance and usability. *Molecular biology and evolution*, *30*(4), 772-780.
Schmidt, H. A., & von Haeseler, A. (2015). IQ-TREE: a fast and effective stochastic algorithm for estimating maximum-likelihood phylogenies. *Molecular Biology and Evolution*, *32*(1), 268-274.
Yu, G., Smith, D. K., Zhu, H., Guan, Y., & Lam, T. T. Y. (2017). ggtree: an R package for visualization and annotation of phylogenetic trees with their covariates and other associated data. *Methods in Ecology and Evolution*, *8*(1), 28-36.
Wang, L. G., Lam, T. T. Y., Xu, S., Dai, Z., Zhou, L., Feng, T., ... & Yu, G. (2020). Treeio: an R package for phylogenetic tree input and output with richly annotated and associated data. *Molecular biology and evolution*, *37*(2), 599-603.

Table S3: Sequences of *Nodularia* species and other Nostocalean species as outgroups for phylogenetic tree construction (suppl. Figure S2 & S3). Primary focus on Baltic species from NCBI. 16S rRNA sequences were aligned using MAFFT based on local pairwise alignment and at maximum 1000 iterations (v7.481) (Katoh & Standley, 2013).

Species	Strain	Accession No	Resource (origin)	Reference	Comment
Aphanizomenon flos-aquae	FACHB-1249	NZ_JACJTM010000096.1	China	10.1038/s41396-020-00775-z	
Aphanizomenon flos-aquae	FACHB-1287	NZ_JACJTO010000084.1	China	10.1038/s41396-020-00775-z	
Aphanizomenon flos-aquae	FACHB-1416	NZ_JACJTP010000161.1	China	10.1038/s41396-020-00775-z	
Dolichospermum lemmermannii	04-24	FN691916.1	Czech Republic:Husinec		
Dolichospermum lemmermannii	FEM_GDL2	LN871456.1	Lake Garda,Italy	10.1016/j.hal.2015.09.008	
Dolichospermum lemmermannii	FEM_CDL3	LN871468.1	Lake Como, Italy	10.1016/j.hal.2015.09.008	
Dolichospermum sp.	FACHB-1091	NZ_JACJSB010000058.1	NA	10.1038/s41396-020-00775-z	
Dolichospermum sp.	LEGE 00240	NZ_JADEVZ010000001.1	Maranho dam reservoir, Portual		
Dolichospermum sp.	UHCC 0259	NZ_VIKX01000317.1	NA	10.3390/toxins12040248	
Nodularia baltica	BY1	AJ133177.1	NA	10.1099/00207713-50-3-1043	
Nodularia harveyana	SAG 44.85	KC912773.1	salt marsh;UK		
Nodularia harveyana	SAG 44.85	KF010323.1	salt marsh;UK		
Nodularia harveyana	CDAC1983/300	AF268020.1	Hiddensee	10.1099/00207713-51-2-505	
Nodularia sp.	NIES-3585	BDUB01000001.1	Japan	10.1093/dnares/dsab024	
Nodularia sp.	20-22 strain 2	MW491279.1	Baltic Sea	10.3389/fmicb.2021.681881	
Nodularia sphaerocarpa	NA	AF268018.1	France	10.1099/00207713-51-2-505	
Nodularia sphaerocarpa	UP16f	AJ133182.1	Baltic Sea (Gulf of Finland)	10.1099/00207713-50-3-1043	
Nodularia sphaerocarpa	HKVV	AJ133183.1	Baltic Sea (Stockholm Archipelago)	10.1099/00207713-50-3-1043	
Nodularia spugimena	AV2	MF680045.1	Baltic Sea	10.1021/acschembio.7b00570	
Nodularia spugimena	HEM	AF268005.1	Baltic Sea	10.1099/00207713-51-2-505	
Nodularia spugimena	NSLA02A4	AF268008.1	Lake Alexandrina	10.1099/00207713-51-2-505	
Nodularia spugimena	NSLA01	AF268009.1	Lake Alexandrina	10.1099/00207713-51-2-505	
Nodularia spugimena	HKVV	AF268024.1	Baltic Sea	10.1099/00207713-51-2-505	
Nodularia spugimena/ N. sphaerocarpa	BY1	AF268004.1	Baltic Sea	10.1099/00207713-51-2-505	
Nodularia spumigena	HEM	MF680044.1	Baltic Sea	10.1021/acschembio.7b00570	
Nodularia spumigena	309	MF680046.1	Baltic Sea	10.1021/acschembio.7b00570	
Nodularia spumigena	PCC 73104	NR_112106.1	Spotted lake, BC, Canada	10.1111/j.1574-6968.2001.tb10736.x	
Nostoc sp.	NIES-2109	LC322124.1	NA		
Nostoc sp.	NIES-2111	LC322125.1	NA		
Trichormus variabilis	SAG 1403-4b	KX014845.1	NA		
Nodularia sp.	1-2105	PQ363512.1	Baltic Sea		short fragment
Nodularia sp.	1-2111	PQ363520.1	Baltic Sea		short fragment
Nodularia sp.	1-2109.2	PQ363513.1	Baltic Sea		short fragment
Nodularia sp.	12-2102	PQ363514.1	Baltic Sea		short fragment
Nodularia sp.	12-2103	PQ363515.1	Baltic Sea		short fragment
Nodularia sp.	12-2101	PQ363516.1	Baltic Sea		short fragment
Nodularia sp.	1-2109.1	PQ363517.1	Baltic Sea		short fragment
Nodularia spumigena	PB	PQ363518.1			short fragment
Nodularia harveyana	SAG 44.85	PQ363519.1			short fragment
Nodularia sp.	12-2102	PQ363521.1	Baltic Sea		long fragment
Nodularia sp.	12-2103	PQ363522.1	Baltic Sea		long fragment
Nodularia sp.	12-2101	PQ363523.1	Baltic Sea		long fragment
Nodularia sp.	1-2109	PQ363524.1	Baltic Sea		long fragment
Nodularia spumigena	PB	PQ363525.1			long fragment
Nodularia harveyana	SAG 44.85	PQ363526.1			long fragment
Synechococcales cyanobacterium	1-2105.1	PQ363527.1	Baltic Sea		long fragment
Nodularia sp.	1-2111	PQ363528.1	Baltic Sea		long fragment

Suppl. Results:

Molecular identity of isolates

To confirm the morphological characterization and therefore the identity of the resurrected *N. spumigena* strains genetically, we blasted the amplified sequences and generated a phylogenetic tree based on 16S rRNA fragments. All strains sequences, except one, showed 98.5 – 99.9% identical sites to *Nodularia* sp. and *Nodularia spumigena* sequences. Our isolated *Nodularia* strains differed by at least three nucleotides from the downloaded *N. harveyana* sequences. Tree reconstruction was created for both short (185 nt) and long (383 nt) fragments (Suppl. Figure S2 & Suppl. Figure S3). Although, the bootstrap support was higher and fewer nodes were collapsed in the long fragment tree, the overall results were similar. In both phylogenetic trees, *N. spumigena* and *N. sphaerocarpa* were clustered into a species complex separated by *N. harveyana*. All *Nodularia* strains were clustered into one group with low support (63) in the short fragment tree and high support (94) in the long fragment tree. The phylogenetic relationship of other Nostocacean clusters remain unresolved, with collapsed nodes in both trees.

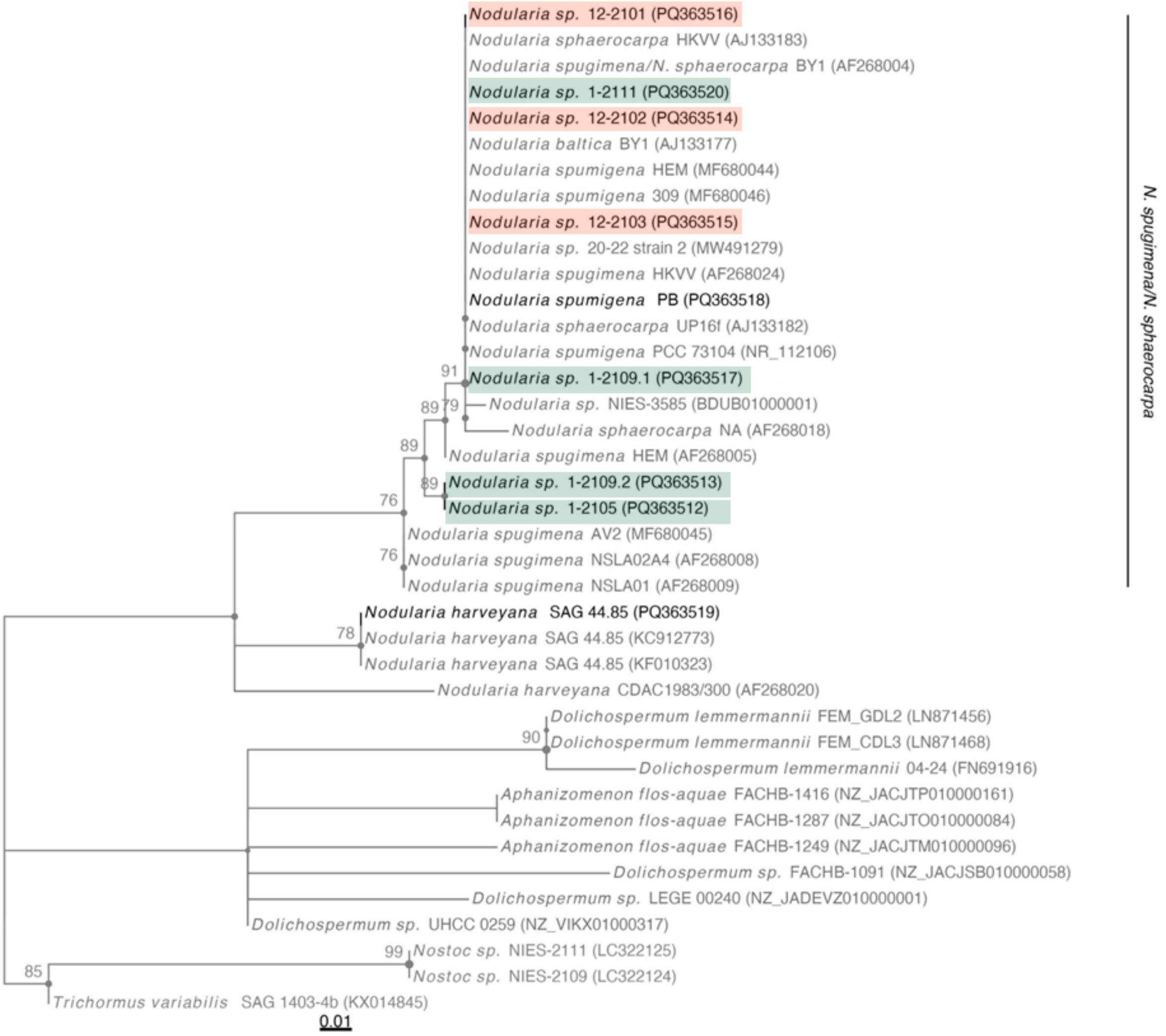

Figure S2*:* Maximum likelihood phylogenetic tree of the cyanobacterial 16S rRNA fragment alignment (185 nt). Isolated and sequenced strains in this study are highlighted in black. Overlayed rectangles highlights the resurrected recent (2020 ± 0.5 CE, green) and subrecent (1987 ± 2 CE orange) strains confirmed as *Nodularia spumigena* (together with morphological identification after Komárek 2013). The GenBank accession numbers are highlighted in parenthesis with each strain. Bootstrap support (1000 replicates) ≥ 70 are shown next to the nodes. Nodes with a bootstrap support < 50 were collapsed. Overlayed rectangle indicate resurrected recent (green) and subrecent (orange) strains.

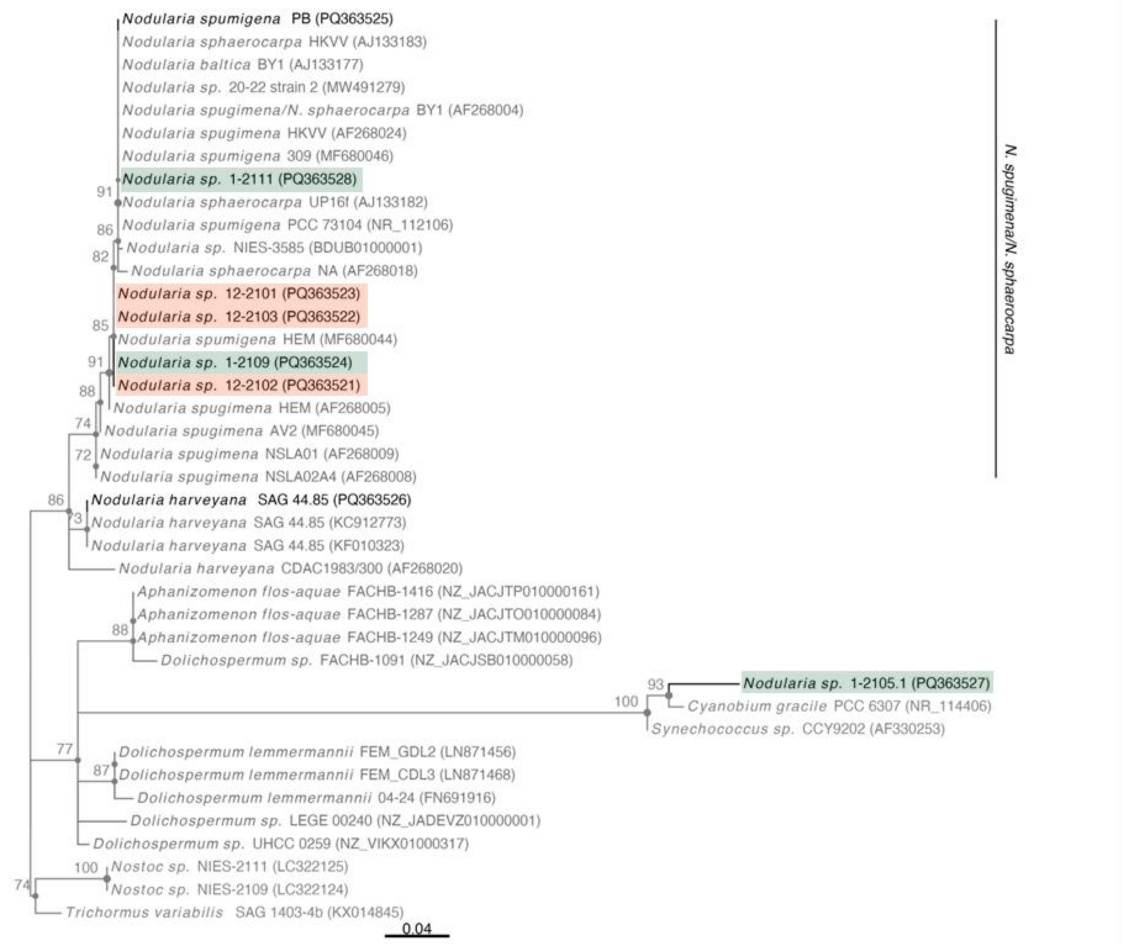

Figure S3*:* Maximum likelihood phylogenetic tree of the cyanobacterial 16S rRNA fragment alignment (383 nt). Isolated and sequenced strains in this study are highlighted in black. and confirmed as *Nodularia spumigena*. Overlayed rectangles highlights the resurrected recent (2020 ± 0.5 CE, green) and subrecent (1987 ± 2 CE orange) strains confirmed as *Nodularia spumigena* (together with morphological identification after Komárek 2013). The GenBank accession numbers are highlighted in parenthesis with each strain. Bootstrap support (1000 replicates) ≥ 70 are shown next to the nodes. Nodes with a bootstrap support < 50 were collapsed. The phylogenetic tree was outgroup-rooted using *Cyanobium*/ *Synechococcus.*

Table S4: Parameters of respective P–I curves (Figure 5) of all resurrected *Nodularia spumigena* strains. Light parameters were modelled after Walsby et al. 1997. Mean ± standard derivation (n = 3), NPP_{max} = netto primary production, Ik = light saturation point, Ic = light compensation point , beta = photoinhibition coefficient (β).

age cohort	strain	NPP_{max} (µmol O_2 mg^{-1} Chl *a* h^{-1})	Respiration (µmol O_2 mg^{-1} Chl *a* h^{-1})	Ik (µmol photons m^{-2} s^{-1})	Ic (µmol photons m^{-2} s^{-1})	alpha	beta
**2020 CE**	**1-2105**	101.8 ± 7.8	-16.3 ± 0.8	132.8 ± 7.9	19.1 ± 3.3	0.9 ± 0.1	-0.02 ± 0.0
	**1-2109**	46.2 ± 5.3	-16.1 ± 2.9	119.1 ± 19.6	35.0 ± 3.1	0.5 ± 0.1	-0.01 ± 0.0
	**1-2111**	78.0 ± 4.9	-13.6 ± 1.7	101.2 ± 4.5	17.0 ± 1.7	0.9 ± 0.1	0.00 ± 0.0
**1987 CE**	**12-1201**	18.7 ± 7.1	-20.0 ± 3.0	91.9 ± 47.3	89.4 ± 30.1	0.4 ± 0.2	0.00 ± 0.0
	**12-1202**	226.6 ± 71.7	-31.3 ± 8.1	154.1 ± 99.8	52.9 ± 9.9	3.9 ± 3.8	-3.31 ± 3.8
	**12-1203**	161.2 ± 43.0	-18.7 ± 4.6	148.0 ± 30.4	16.7 ± 1.6	1.2 ± 0.2	-0.01 ± 0.0

Table S5: Results of cell size measurments of the resurrected *Nodularia spumigena* strains kept at 15°C and 30 – 40 µmol photons m^{-2} s^{-1}. L = cell lengths, W = cell width in µm ± standard derivation (SD) (n = 20).

		vegetative cells		heterocysts		akinetes	
**age cohort**	**strain**	**L± SD**	**W± SD**	**L ± SD**	**W ± SD**	**L ± SD**	**W ± SD**
2020	**1-2105**	3.2 ± 0.6	9.2 ± 0.8	4.2 ± 0.4	10.1 ± 1.1	8.5 ± 1.7	12.0 ± 1.2
	**1-2109**	2.7 ± 0.4	9.6 ± 0.7	3.8 ± 0.8	8.7 ± 0.9	7.4 ± 0.9	9.8 ± 0.9
	**1-2111**	3.3 ± 0.7	9.2 ± 0.6	4.4 ± 0.9	10.3 ± 0.7	7.9 ± 1.5	12.2 ± 1.0
1987	**12-2101**	3.7 ± 0.5	9.2 ± 0.6	3.9 ± 0.7	10.5 ± 0.7	6.0 ± 0.8	10.6 ± 1.1
	**12-1202**	3.2 ± 0.7	13.1 ± 0.7	4.9 ± 0.8	13.9 ± 1.4	9.4 ± 1.9	14.6 ± 2.6
	**12-2103**	3.3 ± 0.5	12.8 ± 0.4	4.3 ± 0.7	13.8 ± 1.2	7.6 ± 1.4	15.6 ± 2.5

Table S6: Results of trait characterization of the resurrected recent (2020 ± 0.5 CE) and subrecent (1987 ± 2 CE) *N. spumigena* strains (Figure 6). Means ± standard derivation of each strain. Y(II) = effective quantum yield of PSII revealed from PAM measurments (n = 4). Chlorophll *a* (Chl *a*) in mg L^{-1}, total Carbon (Ct), total Nitrogen (Nt) content in pg mg^{-1} dry weight (DW = dry weight), Carbon Nitrogen ratio (n = 3).

age cohort	strain	Y(II)	Chl *a* (mg L^{-1})	N_t (pg mg^{-1} DW)	C_t (pg mg^{-1} DW)	C:N ratio
	**1-2105**	0.357 ± 0.023	0.884 ± 0.067	0.066 ± 0.005	0.351 ± 0.011	5.359 ± 0.289
**2020 CE**	**1-2109**	0.370 ± 0.017	0.609 ± 0.054	0.051 ± 0.003	0.261 ± 0.006	5.112 ± 0.289
	**1-2111**	0.291 ± 0.010	0.678 ± 0.115	0.067 ± 0.002	0.282 ± 0.005	4.237 ± 0.224
	**12-2101**	0.259 ± 0.021	0.725 ± 0.039	0.050 ± 0.003	0.218 ± 0.012	4.338 ± 0.294
**1987 CE**	**12-1202**	0.182 ± 0.007	0.109 ± 0.005	0.021 ± 0.002	0.084 ± 0.015	3.989 ± 0.469
	**12-2103**	0.270 ± 0.032	0.298 ± 0.025	0.032 ± 0.002	0.149 ± 0.004	4.637 ± 0.252
